# Characteristics of Trials Preceding FDA Approval of Novel Psychiatric Drugs

**DOI:** 10.1001/jamanetworkopen.2024.56588

**Published:** 2025-01-27

**Authors:** Rosa Y. Ahn-Horst, Erick H. Turner, Aaron S. Kesselheim

**Affiliations:** 1 Program on Regulation, Therapeutics, and Law (PORTAL), Division of Pharmacoepidemiology and Pharmacoeconomics, Brigham and Women’s Hospital, Boston, Massachusetts; 2 Department of Psychiatry, Massachusetts General Hospital, Boston; 3 Department of Psychiatry, McLean Hospital, Belmont, Massachusetts; 4 Harvard Medical School, Boston, Massachusetts; 5 Department of Psychiatry, Oregon Health & Science University, Portland

## Abstract

This cross-sectional study reports on the study design characteristics of clinical trials for novel psychiatric drugs approved by the US Food and Drug Administration.

## Introduction

Mental illnesses are among the most common and disabling diseases with substantial unmet need.^1^ Unlike many other fields of medicine, psychiatric diagnoses lack validated biomarkers,^2^ and relatively little is known about the pathophysiology of psychiatric disorders,^3^ posing major challenges in drug development.^4^ We evaluated psychiatric drug approvals in the past decade to understand the regulatory background of new psychiatric medications and the evidence on which they were approved.

## Methods

This cross-sectional study examined all novel drugs approved by the US Food and Drug Administration (FDA) to treat psychiatric indications between January 2013 and October 2024. Novel drugs included new molecular entities (NME), new active ingredients, and reformulated drugs for which efficacy trials were conducted (during the study period), each of which received FDA approval for their first psychiatric indication.^5^ We included drugs targeting mood, behavioral, attention-deficit/hyperactivity, and psychotic disorders. This study followed the Strengthening the Reporting of Observational Studies in Epidemiology (STROBE) reporting guideline.

We used Drugs@FDA, a publicly accessible database, to obtain regulatory characteristics for each drug.^5^ We also extracted study participant demographics and key trial design features (randomization, masking, comparator, primary trial end point, and trial duration) on pivotal clinical trials leading to each drug’s approval. We categorized pivotal trial results as positive or not based on the FDA reviewer’s assessment. In addition to pivotal trials, we included all other efficacy trials submitted to the FDA as supportive studies and extracted primary end point results. We used descriptive statistics to characterize all results. Data were analyzed with STATA version 18 (StataCorp LLC).

## Results

Between 2013 and 2024, the FDA approved 16 novel drugs to treat psychiatric diseases (Table 1). The mechanism of action for these drugs involved a combination of serotonin, norepinephrine, and/or dopamine systems (11 drugs), N-methyl-D-aspartate (1 drug), γ-aminobutyric acid (2), α-2 adrenergic (1 drug), and muscarinic acetylcholine receptors (1 drug).

**Table 1.** Novel Psychiatric Drugs Approved by the US Food and Drug Administration (FDA), 2013-2024

Drug name, by indication	Year approved	Mechanism of action	Special FDA designation	Clinical testing duration (IND to NDA), y	Positive trials/total efficacy trials submitted, No. (%)	Positive pivotal trials, No.^a^	Pivotal trial primary end point^b^	
MDD								
Levomilnacipran	2013	Inhibition of serotonin/NE reuptake	None	3.6	4/6 (67)	3		MADRS
Vortioxetine	2013	Inhibition of serotonin uptake	None	5.4	6/10 (60)	6		MADRS; HAMD-24
Gepirone	2023	5-HT_1A_ agonist	None	17.7	2/12 (17)	2		HAMD-17
Treatment-resistant depression								
Esketamine	2019	NMDA antagonist	Fast track; priority review; breakthrough	6.3	3/5 (60)	2		MADRS; time to relapse using MADRS, or hospitalization
Postpartum depression								
Brexanolone	2019	GABA_A_ modulator	Breakthrough; priority review	3.8	3/3 (100)	3		HAMD
Zuranolone	2023	GABA_A_ modulator	Breakthrough; priority review; fast track	6.2	2/2 (100)	2		HAMD-17
Adjunctive therapy for MDD								
Brexpiprazole	2015	Partial 5-HT_1A_, D_2_ agonist; 5-HT_2A_ antagonist	None	6.3	1/4 (25)	1		MADRS
Schizophrenia								
Brexpiprazole	2015	Partial 5-HT_1A_, D_2_ agonist; 5-HT_2A_ antagonist	None	6.3	2/3 (67)	2		PANSS
Aripiprazole lauroxil	2015	Partial 5-HT_1A_, D_2_ agonist	None	3.9	1/1 (100)	1		PANSS
Lumateperone	2019	5-HT_2A_ and D2 antagonist	Fast track	10.8	2/3 (67)	2		PANSS
Olanzapine/samidorphan^c^	2021	Dopamine and 5-HT_2_ antagonism; opioid receptor antagonism	None	12	3/3 (100)	2		PANSS; percentage change in body weight
Xanomeline tartrate and trospium chloride	2024	Muscarinic agonist; muscarinic antagonist	None	8.2	3/3 (100)	2		PANSS
Cariprazine	2015	Partial 5-HT_1A_, D_2_ agonist; 5-HT_2A_ antagonist	None	7.4	3/4 (75)	3		PANSS
Bipolar disorder^d^								
Cariprazine	2015	Partial 5-HT_1A_, D_2_ agonist; 5-HT_2A_ antagonist	None	7.4	3/3 (100)	3		YMRS
ADHD								
Serdexmethylphenidate/dexmethylphenidate	2021	Inhibition of NE/dopamine reuptake	None	3.5	1/1 (100)	1		SKAMP-C
Viloxazine	2021	Inhibition of NE reuptake	None	10.4	3/4 (75)	3		ADHD-RS-5
Parkinson disease psychosis								
Pimavanserin	2016	5-HT_2A_ antagonist	Priority review; breakthrough	11.8	1/4 (25)	1		SAPS-PD
Acute agitation associated with schizophrenia or bipolar disorder								
Dexmedetomidine	2022	Alpha-2 adrenergic agonist	Fast track	2.3	2/2 (100)	2		PEC

Abbreviations: ADHD, attention-deficit/hyperactivity disorder; ADHD-RS-5, ADHD Rating Scale-5; GABA, γ-aminobutyric acid; HAMD, Hamilton Rating Scale for Depression; 5-HT, 5-hydroxytryptamine (serotonin); IND, investigational new drug; MADRS, Montgomery-Ashberg Depression Rating Scale; MDD, major depressive disorder; NDA, new drug application; NE, norepinephrine; NMDA, N-methyl-D-aspartate; PANSS, Positive and Negative Syndrome Scale; PEC, PANSS-Excited component; SAPS-PD, Scale for the Assessment of Positive Symptoms adapted for Parkinson Disease; SKAMP-C, Swanson, Kotkin, Agler, M-Flynn, and Pelham-Combined; YMRS, Young Mania Rating Scale.

^a^ Positive trials are not necessarily considered to be pivotal by the FDA if they are not deemed to be essential for approval (eg, phase 2 trials).

^b^ Multiple primary end points are listed when more than one pivotal trial exists for a given indication, and the pivotal trials have different primary end points from one another.

^c^ Did not include its second indication for bipolar disorder (acute treatment of manic or mixed episodes as monotherapy and as adjunct to lithium or valproate) because olanzapine/samidorphan was tested only in the schizophrenia population, as the FDA had “no reason to believe efficacy of olanzapine would be uniquely impaired by samidorphan in people with bipolar 1 disorder.”

^d^ Acute treatment of manic or mixed episodes associated with bipolar disorder.

The FDA approvals in this sample were associated with 73 clinical trials, of which the FDA judged 45 (62%) to be positive. There were 3 drugs for which fewer than half the submitted efficacy trials were positive: brexipiprazole (3 of 7 [43%]), pimavanserin (1 of 4 [25%]) (a drug deemed not approvable by the FDA medical reviewers whose decision was overturned by leadership following a favorable advisory committee vote), and gepirone (2 of 12 [17%]), a drug that was rejected thrice before gaining approval.

Among the 73 clinical trials, 46 were designated by FDA as pivotal trials (median 3 pivotal trials/drug [range, 1-6]) (Table 2). Three drugs (19%) were approved after a single pivotal trial. Among pivotal trial participants, there was a similar representation of female (8700 [48.9%]) and male participants (9099 [51.1%]); 946 patients were Asian (5.3%), 5328 were Black (30.0%), and 10 884 were White (61.2%). All pivotal trials were randomized and double-masked. Most trials were placebo-controlled (38 [83%]) and another 7 trials (15.2%) had both placebo and active controls, with clinical scales (45 [97.8%]) defining the primary end point. The 3 clinical scales most commonly used were the Positive and Negative Syndrome Scale (PANSS) (12 trials [26%]), the Montgomery-Asberg Depression Rating Scale (MADRS) (12 trials [26%]), and the Hamilton Depression Scale (7 trials [15%]).

**Table 2.** Characteristics of Pivotal Trials of Novel Psychiatric Drugs

Characteristics	No. (%)
Total pivotal trial, No.	46
Positive pivotal trials	41 (89.1)
Pivotal trials per drug, median (range)	3 (1-6)
Total drugs, No.	16
Total participants, median (IQR)	392 (238-568)
Sex	
Female	8700 (48.9)
Male	9099 (51.1)
Race^a^	
American Indian or Alaska Native	95 (0.5)
Asian	946 (5.3)
Black	5328 (30.0)
Native Hawaiian and other Pacific Islander	21 (0.1)
White	10 884 (61.1)
Multiple races	104 (0.6)
Other^b^	404 (2.3)
Comparator	
Active	1 (2.2)
Placebo	38 (82.6)
Placebo and active control	7 (15.2)
Masking	
Double	46 (100)
Single	0
Open label	0
Primary trial end point	
Clinical scales	45 (97.8)
Other	1 (2.0)
Trial duration, median (IQR), wk	6 (4-8)

^a^ Values are for studies with available information only.

^b^ Other includes patients with unknown and not reported race.

## Discussion

In the past decade, a limited number of psychiatric drugs have been approved, and most of their mechanisms of action—involving serotonin, dopamine, and/or norepinephrine systems—are similar to drugs developed decades ago.^6^ Although most drugs were supported by multiple positive phase 3 trials, 3 had more negative or failed efficacy trials than positive ones, including 1 which was approved based on 2 positive trials in the context of 10 other negative or failed trials. Notably, only 1 of these 3 drugs was designated for expedited review. Limitations include results that may not be generalizable to drugs targeting other diseases (eg, movement and substance use–related disorders) and inclusion of prodrugs and drug combinations if at least one drug was an NME. The quality of evidence supporting psychiatric drug approvals varied substantially, underscoring the need for increased clarity and consistent application of FDA approval standards among drugs treating mental illnesses.

## Notes

Supplement.
Data Sharing Statement
