# Supplementary material for: Characteristics of Trials Preceding FDA Approval of Novel Psychiatric Drugs
Source: JAMA Netw Open. 2025 Jan 27;8(1):e2456588. doi: 10.1001/jamanetworkopen.2024.56588 (PMC11774090; doi:10.1001/jamanetworkopen.2024.56588)
Supplement: Supplement. — Data Sharing Statement [file jamanetwopen-e2456588-s001.pdf]

Data Sharing Statement

Ahn-Horst. Characteristics of Trials Preceding FDA Approval of Novel Psychiatric Drugs. *JAMA Netw Open*. Published January 27, 2025. doi:10.1001/jamanetworkopen.2024.56588

Data
Data available: No

Additional Information
Explanation for why data not available: All data used in this paper – extracted from FDA review documents (Drugs@FDA, https://www.accessdata.fda.gov/scripts/cder/daf/index.cfm) – are publicly accessible.
